# The effect of inadequate access to healthcare services on emergency room visits. A comparison between physical and mental health conditions

**DOI:** 10.1371/journal.pone.0202559

**Published:** 2018-08-23

**Authors:** Nerina Vecchio, Debbie Davies, Nicholas Rohde

**Affiliations:** 1 Griffith Business School, Griffith Health Institute, Griffith University, Gold Coast, Australia; 2 Gold Coast Primary Health Network, Gold Coast, Australia; 3 Griffith Business School, Griffith University, Gold Coast, Australia; Yokohama City University, JAPAN

## Abstract

This paper estimates the influence of inadequate access to healthcare services on the rate of Emergency Room (ER) hospital visits in Australia. We take micro-data on different types of healthcare shortfalls from the 2012 Australian Survey of Disability, Aging and Carers, and employ Propensity Score Matching (PSM) techniques to identify their effects on ER visits. We find that shortfalls in access to various medical services increases ER visits for individuals with mental and physical conditions by about the same degree. Conversely, inadequate community care services significantly predict ER visits for individuals with physical conditions, but not for persons with mental conditions. The lack of predictive power for inadequate community care for persons with mental health problems is surprising, as “acopia” is thought to be a significant driver of crises that require emergency treatment. We discuss some of the mechanisms that may underpin this finding and address the policy implications of our results. Lastly a number of robustness checks and diagnostics tests are presented which confirm that our modelling assumptions are not violated and that our results are insensitive to the choice of matching algorithms.

## Introduction

Many hospitals in developed countries are experiencing increasing pressure due to rising numbers of patient presentations and Emergency Room (ER) admissions [1]. The causes of life threatening or critical conditions that require urgent attention at ERs are numerous and include patient-related, illness-related and system related factors [2]. Compounding the pressure that these factors place on ER admission rates is inadequate access to health care services in the community, which can lead undertreated health conditions to escalate to critical levels [3]. Furthermore, statistics show that many ER presentations are non-urgent and often do not require specific hospital treatment [4, 5]; [6]. Long consultations by GPs provide preventative care and chronic disease management. In Australia the drastic decline in long consultations in preference for short consultations [7] may be adding to patient perception that the GP is not able to deal with their health issue. This implies that individuals with non-urgent health matters substitute inaccessible health care services for ER services [8, 9]. Consequently it is likely that the under provision of health care services in the community is leading to an excessive and suboptimal burden on ER systems.

The main objective of this study is to estimate the influence of an individual’s perceived ability to access healthcare services on ER presentation rates. Specifically, in this paper inadequate health care services is defined as a situation where a person requires more assistance than is currently being received for services where assistance is needed. Obtaining a detailed understanding of the determinants of ER admissions is desirable as it allows policy makers to better tailor the provision of limited healthcare services. This would allow for improved access for at-risk individuals, and would have the effect of reducing instances where easily treatable health conditions deteriorated into medical emergencies. As medical emergencies represent a poor outcome for the individual (and are costly for governments to finance) better targeting of healthcare services has the capacity to both improve public health and save on health related expenditure.

In order for this type of analysis to be effective it is necessary to determine what types of healthcare shortfalls best predict ER admission rates, and to identify the types of individuals who are most at risk. For example some persons may lack the ability to obtain specific medical services such as that provided by GPs (or by the non-emergency departments of hospitals) while others may not receive appropriate community care for ongoing conditions. Establishing which forms of shortfall are most important and how these effects can vary by disabling condition provides information on how best to allocate funding for healthcare services. If either a distinct group of patients or a type of health service can be identified then specific intervention strategies can be directed towards modifiable factors that reduce ER rates. This will create opportunities to develop cost effective strategies that reduce waiting times and improve health outcomes.

A second focus of the paper involves studying some unexpected results we uncover that are specific to persons experiencing inadequate community care. While all other forms of healthcare shortfall predict increases in ER visits, we do not find significant associations between some of these variables for individuals with mental health issues as their primary disabling condition. Such a finding is counterintuitive as persons with mental conditions are more likely than the general population to experience “acopia” (i.e. self-neglect due to excessive psychosocial stress) or choose not to participate in a health plan (i.e. noncompliance with medications) which could plausibly (i) result in ER visits, and (ii) be mitigated by suitable access to community care. A number of plausible explanations for this lack of empirical association exist, each of which has important and differing implications for the distribution of resources for people with mental health conditions.

The rest of the paper is structured as follows. Section 2 provides some background information and reviews the literature on shortfalls in healthcare services and on the determinants of ER visits in developed countries. Section 3 introduces our data set, describes our analytical approach and presents the results. Section 4 interprets the findings and Section 5 concludes.

## Background

There is an extensive body of literature that studies the determinants of ER admissions in developed countries, and presentations have generally been attributed to a combination of factors. Padgett [10] proposes that use of the ER is the consequence of predisposing (e.g. age, sex, ethnicity, education, psychosocial resources and attitudes about health care), enabling (e.g. insurance coverage and income), and need factors (e.g. measures of health status and evaluations of need for health care, socioeconomic stress, psychiatric co-morbidities, lack of social support).

A major modifiable factor linked to the use of ER services is the absence of primary care. Research into inadequate service access is frequently motivated by the notion that it forms a proxy measure for the quality of community health care programmes and programme efficacy [11, 12]. The greatest amount of attention by researchers regarding the link between ER use and service availability relates to GP services [9, 13–16]. Studies show that the high level of utilization of ER services reflect problems relating to GP services including accessibility, affordability and inability to provide appropriate diagnosis [6, 8, 17]. Ingram [18] shows that the actual or perceived unavailability of the physician is an important factor in the use of hospital emergency facilities. Patients with non-urgent complaints generally reside within close proximity of the hospital providing evidence that the emergency facilities may act as an “off-hours” physician surrogate [18]. Studies by Kravet et al [9], 2008) and Wright et al [16] find a higher primary care physician ratio in an area associated with a statistically significant decrease in ER visits.

An integral component of primary health care is the home and community care program that aim to provide a comprehensive, coordinated and integrated range of basic maintenance and support services to assist people to live independently at home. Although community care is central to an integrated health care system (Australian Council on Healthcare Standards, 2010; Goddard *et al*., 2000) these programs remain under researched in ER investigations. This is primarily due to limited information that has encouraged researchers to focus on GP services [9, 13–16].

Appropriate access to healthcare services are an integral part of Western health care systems. Given the increasing integration of health care programs, an investigation of ER use that explores a range of health care services (rather than GP services alone) is warranted. There are numerous ways to categorize the forms that these may take, and for simplicity in this study we stratify by community care services such home nursing and allied health support or personal assistance, and external expert medical services such as those provided by GPs or by the non-emergency facilities at hospitals. Furthermore, investigations consistently report the inaccessibility of health care services among people experiencing mental health conditions [19–21]. This difference in access to health care resources can play an influential role in ER presentations. Yet studies typically do not control for major categories of health conditions (see Tang [22]).

## Methods and results

### Data

Data for our study come from the 2012 wave of the Survey of Disability, Aging and Carers [23]. This is a large, nationally representative micro-data set compiled by the Australian Bureau of Statistics that records observations on a wide variety of questions related to various aspects of health and social behaviour. Despite the size of the survey (there are almost around 80,000 individuals surveyed and several thousand questions asked in the latest edition) it has not been widely used in applied work. Due to data limitations relating to service delivery, the sample was confined to recipients of care aged 20 years and over, residing in the community. Descriptive statistics of our sample are given in Table 1. As expected our data primarily cover older people, with a lower than average rate of educational attainments. Females are slightly over-represented and most of our sample resides in major cities.

**Table 1:** Descriptive statistics–key variables.

Variable	Mean	Std Dev	Min	Max
ER Visits	0.230	0.421	0	1
Activities P	0.022	0.148	0	1
Activities M	0.005	0.070	0	1
Health Conditions P	0.113	0.316	0	1
Health Conditions M	0.012	0.108	0	1
GP Services P	0.151	0.358	0	1
GP Services M	0.031	0.172	0	1
Hospital Services P	0.113	0.316	0	1
Hospital Services M	0.012	0.108	0	1
Female	0.535	0.499	0	1
Age				
20–29	0.052	0.222	0	1
30–39	0.057	0.232	0	1
40–49	0.098	0.297	0	1
50–59	0.139	0.346	0	1
60–69	0.270	0.444	0	1
70–79	0.248	0.432	0	1
Education				
Degree/Dip	0.268	0.443	0	1
Cert/yr 12	0.508	0.500	0	1
Income	4.736	2.176	1	10
Marriage	0.528	0.499	0	1
States				
VIC	0.208	0.406	0	1
QLD	0.165	0.371	0	1
SA	0.140	0.347	0	1
WA	0.104	0.306	0	1
TAS	0.074	0.262	0	1
NT	0.020	0.141	0	1
ACT	0.051	0.220	0	1
Region				
City	0.624	0.485	0	1
Regional	0.238	0.426	0	1
Born in Aust	0.841	0.365	0	1
Welfare Recpt	0.692	0.461	0	1
Disability				
Profound	0.095	0.293	0	1
Severe	0.113	0.316	0	1
Moderate	0.139	0.345	0	1
Mild	0.275	0.447	0	1
Restricted	0.053	0.223	0	1
Not restricted	0.094	0.291	0	1

The table gives descriptive statistics for the variables used in our estimations. Means are reported in the first column while standard deviations are in the second. The last two columns give variable minimums and maximums.

Our main variable of interest is a dummy variable indicating whether or not an individual has visited a hospital for emergency treatment in the previous 12 months. This variable includes both self-visits and those initiated by healthcare providers, such as visits via the ambulance service. Although it is possible to construct count data on the number of ER visits, since the data is mostly characterized by zeros and ones (i.e. most people have either no visits or only one) we simplify by only recording the presence of one or more ER visits. This is done due to complexities in modelling count data that are highly skewed with excess zeros. However an implication of this modelling process is that it prevents us from detecting instances of repeated ER usage, which leaves one potential source of inefficiency unexplored.

In order to contrast the effects on ER visits of different types of healthcare shortfalls, we take a basket of four indicators that capture alternative facets of exposure–organised services that assist with daily activities, organised services that assist with health conditions, GP services and hospital services. Organised services that assist with health conditions perform tasks such as: foot care, taking medication, administering injections, dressing wounds, using medical machinery, manipulating muscles or limbs. Organised services that assist with daily activities perform one or more of the following tasks: cognition or emotion, communication, health care, housework, meal preparation, mobility, paperwork, property maintenance, self-care, transport. These organised services are performed in the community, primarily in the home. Hospital services include inpatient and outpatient services such as elective care, covering medical, surgical and maternity services. The category also covers hospital admissions including cases where patients are not required to stay overnight. Hospital services category excludes hospital emergency department visits.

An individual experiences a shortfall in service provision if they report a perceived need for access to more of a particular type of service. The two variables relating to organised services—frequency of need for more assistance with activities from organised services; frequency of need for more assistance with health care from organised services—were converted to a dichotomous variable. GP services variable was derived from unmet need for GP services in the last 12 months. Our variables all take the form of dummies and inadequacy is defined by the individual relative to their perceived need. Each is therefore reliant upon self-assessments and the usual caveats about subjective measurements in health therefore apply. We argue however that since there are both objective and subjective barriers that can limit healthcare access, self-assessments make the most appropriate method for summarising accessibility.

In addition we take a large number of control variables that can account for extraneous factors that can explain differences in ER visits. The choice of variables are based on a widely used behavioural model of health services utilization developed by Andersen and Newman [24] and refined by Padgett to apply specifically to the use of the ER [10]. These variables include age, gender, income, education level and marital status of the individual, location, measures of population density and dummies that can account for the presence and severity of a medical condition. Lastly as we wish to differentiate between the effects of these shortfalls on mental and physical conditions we also take observations on the form of the main disabling condition of an individual. Mental conditions include: Mental and behavioural disorders, dementia, schizophrenia depression/mood affective disorders, phobic and anxiety disorders, nervous tension/stress, intellectual and developmental disorders, mental retardation/intellectual disability, autism and related disorders, attention deficit disorder/hyperactivity, speech impediment, other mental and behavioural disorders, alzheimer's disease. Main condition is defined by the Australian Bureau of Statistics as a long-term health condition identified by a person as the one causing the most problems. Where only one long-term health condition is reported, this is recorded as the main long-term health condition [23]. Missing and non-conforming data are dropped which leaves us with a final sample of approximately 12,000 individual observations.

### Methods

To analyse the effects of healthcare shortfalls on ER visits we begin with a simple description of the data. Table 2 gives the visit rates (calculated as average proportion of people who visit the ER per year) for persons who did and did not receive adequate healthcare services, and the results are stratified by condition. The first row of Table 2 gives baseline rates for individuals who have no need for care or experience adequate healthcare. These persons experienced suitable care within the community and had ER visit rates of about 21% to 23% per year. In contrast the ER rates are higher among individuals who report inadequate access to services. For individuals with a health condition that is inadequately addressed the rates are much higher (33%). The first two columns under ‘Help with Activities’ show the visit rates for persons lacking sufficient community care are about 10% higher than the baseline group, with slightly greater estimates when the shortfall in care is for an individual with a physical condition compared to a mental condition. For individuals with limited access to medical services the admission rates increase more. Specifically, persons lacking adequate access to a GP have rates 11–14% higher than this baseline while persons unable to obtain adequate access to hospital services had more than double the raw probability of admission of the baseline group.

**Table 2:** Raw differences in emergency room rates by healthcare services—population subgroups^a^.

	Help With Activities		Help with Health Conditions		GP Services		Hospital Services	
	PH	MH	PH	MH	PH	MH	PH	MH
Adequate	0.209	0.226	0.216	0.229	0.213	0.226	0.223	0.229
Inadequate	0.328	0.331	0.339	0.326	0.327	0.366	0.547	0.508
Difference	0.119***	0.105***	0.123***	0.097***	0.114***	0.140***	0.324***	0.279***

^**a**^ Each column gives the ER visit rates for individuals with and without adequate healthcare. Hypothesis tests on differences are conducted with t-statistics and robust standard errors.

*, ** and *** denote 10%, 5% and 1% significance respectively. PH and MH denote shortfalls in health care services for persons with physical and mental conditions.

Despite their clarity, the absolute differences in visit rates given above cannot be interpreted as the causal effect of shortfalls in healthcare services as individuals who are exposed to these shortfalls may differ systematically from those who are not. Indeed it is plausible that persons who miss out on adequate health services may have different propensities to require emergency care than those whose care is adequate, aside from the direct effect generated by the gap in medical attention. For this reason there is a need to control for the presence of potentially confounding factors that may explain ER visits. We employ two alternative methods for controlling for these factors. The first are regression models which are attractive in their simplicity, and are convenient in that they highlight the relationships between all the covariates and the dependant variable. The second set of methods use Propensity Score Matching (PSM) techniques to try to identify the causal effects of our variables of interest.

We consider the regression models first. Let *y* denote an indicator variable that is equal to one if an individual makes an emergency visit within a 12 month period, and let *D* denote a dummy that identifies individuals who experienced a shortfall in access to healthcare. A simple method for estimating the effect of *D* on *y* while controlling for covariate matrix *X* is to estimate the linear probability model

y=Xβ+δD+ε (1)

where *δ* measures the difference in average rates of admission attributable to *D*. This specification is appropriate for estimating *δ* (although not for prediction) and we prefer it to other models such as logits or probits as the linear specification estimates the effect of each shortfall as a constant averaged over the sample, which eases interpretation considerably. If a non-linear binary choice model was used we would have to calculate the effect as experienced by a representative individual rather than the full sample. Estimated parameter values are given in Table 3, while inference is performed with White [25] heteroskedastic standard errors.

**Table 3:** Determinants of ER visits by healthcare services–linear probability models ^a^.

Variable	Help with Activates		Help with health conditions		GP services		Hospital services	
	PH	MH	PH	MH	PH	MH	PH	MH
Constant	0.128***	0.129***	0.126***	0.129***	0.129***	0.129***	0.132***	0.129***
Female	-0.003	-0.001	-0.002***	-0.001***	-0.003	-0.001	-0.002	-0.001
Age								
20–29	0.094***	0.083***	0.103***	0.085***	0.073**	0.074***	0.080***	0.082***
30–39	0.106***	0.098***	0.118***	0.100***	0.086**	0.092***	0.091***	0.096***
40–49	0.041**	0.036**	0.054**	0.038**	0.022	0.031*	0.027	0.035**
50–59	0.023	0.019	0.035	0.020	0.006	0.016	0.014	0.018
60–69	-0.002	-0.005	0.005	-0.005	-0.010	-0.006	-0.007	-0.005
70–79	0.000	-0.002	0.004	-0.001	-0.004	-0.002	-0.003	-0.002
Education								
Degree/Dip	0.009	0.008	0.008	0.008	0.008	0.008	0.008	0.008
Cert/yr 12	-0.003	-0.004	-0.004	-0.004	-0.003	-0.004	-0.004	-0.004
Income	0.003	0.003	0.003	0.003	0.002	0.003	0.003	0.003
Marriage	-0.017**	-0.019**	-0.018**	-0.019**	-0.019**	-0.018**	-0.017**	-0.018**
States								
VIC	-0.015	-0.014	-0.014	-0.014	-0.015	-0.014	-0.014	-0.014
QLD	-0.003	-0.002	0.000	-0.002	-0.003	-0.002	-0.003	-0.002
SA	0.000	0.000	-0.001	0.000	0.000	0.000	0.000	0.000
WA	0.033**	0.035**	0.036**	0.035**	0.033**	0.035**	0.034**	0.035**
TAS	-0.013	-0.010	-0.011	-0.010	-0.012	-0.010	-0.010	-0.010
NT	0.064**	0.067**	0.068**	0.067**	0.067**	0.066	0.067**	0.066**
ACT	0.036*	0.034*	0.037*	0.035*	0.035*	0.034	0.035*	0.034*
Region								
City	-0.039***	-0.038***	-0.038***	-0.037***	-0.035***	-0.038***	-0.037***	-0.038***
Regional	-0.032**	-0.032**	-0.032**	-0.031**	-0.030**	-0.033**	-0.032**	-0.032**
Born in Aust	0.005	0.006	0.003	0.006	0.004	0.005	0.005	0.005
Welfare Recpt	0.037***	0.038***	0.038***	0.039***	0.040***	0.037***	0.036***	0.038***
Disability								
Profound	0.174***	0.196***	0.171***	0.197***	0.192***	0.197***	0.189***	0.197***
Severe	0.149***	0.173***	0.152***	0.174***	0.163***	0.173***	0.163***	0.174***
Moderate	0.099***	0.115***	0.098***	0.115***	0.104***	0.115***	0.108***	0.116***
Mild	0.068***	0.074***	0.064***	0.074***	0.072***	0.073****	0.073***	0.073***
Restricted	0.063***	0.069***	0.059***	0.069***	0.067***	0.069***	0.067***	0.068***
Not restricted	0.015	0.018	0.010	0.018	0.016	0.019	0.019	0.019
Inadequate Mental		0.021		0.008		0.081***		0.207***
Physical	0.061***		0.072***		0.083***		0.258***	
*R*^2^	0.042	0.040	0.042	0.041	0.044	0.045	0.048	0.041
*F*	17.96	17.15	17.92	17.15	18.73	17.48	19.87	17.50

^**a**^ Hypothesis tests on differences are conducted with t-statistics and robust standard errors.

*, ** and *** denote 10%, 5% and 1% significance respectively. PH and MH denote shortfalls in health care services for persons with physical and mental conditions. Age referent is 80 years and over. Education referent is year 11 and below. State referent is NSW. Region referent is outer regional and remote. Disability referent is has a long term health condition without a disability.

The results depicted in Table 3 correspond closely to expectations. Our findings show that older unmarried individuals have higher expected rates of visits while individuals living in more densely populated areas had lower rates. Persons with more severe conditions were more likely to visit the ER however there was little evidence that other factors such as gender, education or income had any effect. It is worth observing that the *R*^2^ statistics are quite low (about 4%) across the models. This is also expected; ER visits are almost always the result of unforseen medical crises which are naturally hard to predict.

The key results in Table 3 are the estimates of *δ* given in the final rows. These estimates show that the two insufficient community care variables significantly increases the visitation rate for persons with physical condition by about 6% and 7% respectively. Conversely neither shortfall predicts admissions for persons with mental health conditions, and hence we conclude that the raw differences in visit rates given in Table 2 are due to extraneous subgroup factors rather than any direct effect of poor healthcare. For the medical service variables we see that the shortfalls significantly predict ER visits for both health concepts. Inadequate access to a GP services raises rates by around 8% for individuals with both physical and mental conditions while trouble utilizing hospital services raises admissions by about 25% for physical conditions and 21% for mental conditions. It is worth observing that these increases are extremely large relative to baseline rates and hence access to this form of health service is critically important in determining ER visits.

A more sophisticated method for controlling for confounding factors (and hence producing a better estimate of a true causal effect) involves looking to match and compare individuals who had similar *ex ante* probabilities of failing to receive sufficient services. By examining the differences in visit rates between otherwise similar individuals, we are able to set up a quasi-experimental framework that can produce alternative estimates of the average effect of a treatment variable (in our case those who report inadequate healthcare) on the individuals that are untreated (those who report adequate health care). Our PSM framework is standard (e.g. [26, 27]), although we employ a number of common variants of the method to ensure that our estimates are not unduly affected by assumptions made in the modelling process. We begin by estimating the propensity score, which is the latent probability of an individual experiencing a shortfall on one of our healthcare criteria. Any binary choice model is suitable for this process however we use a standard probit model for the sake of simplicity. Letting *S* denote a shortfall and *ϕ*(.) the Cumulative Distribution Function (CDF) from the normal density, we estimate the following model via maximum likelihood.

P(S=1|X)=ϕ(Xβ) (2)

The fitted value $\hat{p}=\phi \left(X\hat{\beta }\right)$ thus represents the latent probability that a given individual will experience a shortfall conditional on the covariates in *X*. Once these scores are obtained, the key is then to find a suitable method for linking individuals with similar values of ${\hat{p}}_{i}$ across the treated (those who report inadequate healthcare services) and untreated (those that report adequate healthcare services) groups. One simple approach is to match each individual from the treated group with an untreated person that has a similar propensity. This is Nearest Neighbour (NN) matching, which is regarded as a baseline method for constructing counterfactual subgroups. A neat advantage of NN matching is that it can be easily generalized into *k*th order matching, where *k* is an integer value indicating the number of matches for each treated observation.

There are a number of other matching methods that are frequently employed in the literature that have the capacity to produce different results to those obtained from NN. We focus on two alternative methods, caliper matching and kernel weighting, although for the sake of brevity we ignore other variants such as methods based on the Mahalanobis distance. Caliper matching has the capacity to improve upon NN as the latter may be prone to making poor matches when no good candidates are available. By specifying a tolerance (caliper) range and considering all possible matches within that range, a large number of potential linkages become possible while ensuring that poor candidates are not employed. Similarly kernel weighting allows for multiple matches–each observation is compared to a weighted average of all possible candidates where the weighting is determined non-parametrically based upon the similarity of scores.

Estimates based upon (i) nearest neighbour PSM matches for *k* taking on values 1, 2 and 3, (ii) caliper matches obtained using calipers of 0.005 and 0.01, and (iii) kernel weighted matches are obtained for each shortfall and health concept and are presented in Table 4. Our choices of uniform (rather than varying) caliper widths were motivated by a desire to retain a consistent estimation across models, and the specific values were selected on the basis of trade-offs between bias reduction and significance. The kernel density estimator used for weighted matching is based upon biweight kernels and employs a fixed plug-in optimal bandwidth selector.

**Table 4:** PSM estimates of effect of inadequate access to healthcare dervices on ER Rates ^a^.

	Help with Activities		Help with health conditions		GP services		Hospital services	
	PH	MH	PH	MH	PH	MH	PH	MH
Regression	0.061***	0.021	0.072**	0.008	0.083***	0.081***	0.258***	0.207***
PSM NN *k* = 1	0.055***	0.023	0.062***	-0.006	0.064***	0.024	0.208***	0.164*
PSM NN *k* = 2	0.072***	0.008	0.058***	-0.007	0.074***	0.063**	0.224***	0.197**
PSM NN *k* = 3	0.069**	0.012	0.071***	-0.004	0.078***	0.067**	0.208***	0.175**
PSM *C = 0*.*005*	0.061***	0.021	0.067***	0.012	0.081***	0.078***	0.259***	0.291***
PSM *C = 0*.*01*	0.060***	0.021	0.069***	0.023	0.081***	0.079***	0.262***	0.224***
PSM Kernel	0.061***	0.037	0.072***	0.065*	0.083***	0.089***	0.289***	0.273***

^**a**^ The rows give the PSM estimates based upon the specified matching algorithm.

NN denotes Nearest Neighbour matching, C denotes caliper width and Kernel indicates non-parametrically weighted comparisons. *, ** and *** denote 10%, 5% and 1% significance respectively.

The estimates presented above represent the focal point of the paper. We see that over a variety of estimation strategies and matching algorithms, the effects of perceived unmet need with respect to formal assistance with activities and health conditions are significant and robust for physical health, but never for mental health. It is worth emphasizing that these estimates control for the full range of factors given in Table 3 including the presence and severity of any existing condition, and therefore imply that a lack of adequate home care acts to increase admission probabilities by a quantitatively meaningful degree for persons with physical conditions.

For perceived unmet need for medical services (that is, GP and hospital) the results are even stronger and apply equally for both mental and physical conditions. Being unable to access a GP increases ER visits rates by 6%-8% over both conditions and all models, indicating that the finding is robust as well as significant. However the most important distinction is for individuals unable to fully access services provided by hospitals. This shortfall in coverage has dramatic consequences for ER visits, raising rates by more than 20% in absolute terms for both mental and physical conditions.

### Diagnostics

There are a number of assumptions that underlie PSM estimators which must be satisfied for our results from Table 4 to be valid. In this section we conduct some diagnostic tests to examine the performance of our models. The first issue we consider is the support structure of the propensity scores for the treated and untreated subgroups. A crucial requirement underpinning PSM estimation is that there must be sufficient overlap in the scores such that observations in one group can be appropriately matched to an equivalent observation in the other. If (for example) there are observations in the treated group that have propensity scores that greatly exceed the range of scores in the untreated group then the quality of the matching process will be adversely affected.

**Fig 1:**
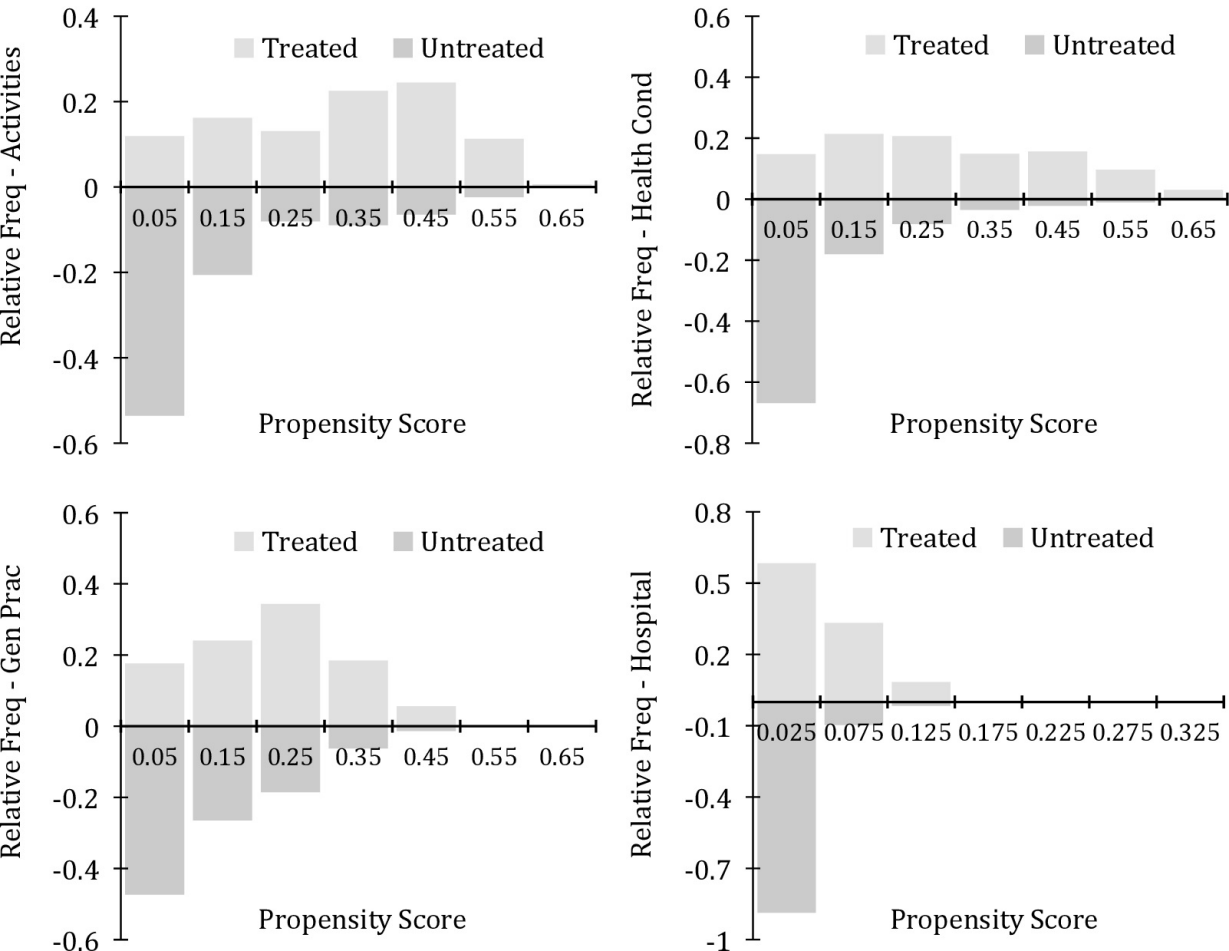
Common support histograms–healthcare shortfalls, physical condition ^a^. ^a^ The vertical axes give the relative frequencies for the propensity scores for each form of healthcare shortfall. The horizontal axes provide the numerical values of the scores. The top left panel represents help with home activities, the top right home healthcare, and the bottom panels give insufficient access to general practitioners and hospital services.

**Fig 2:**
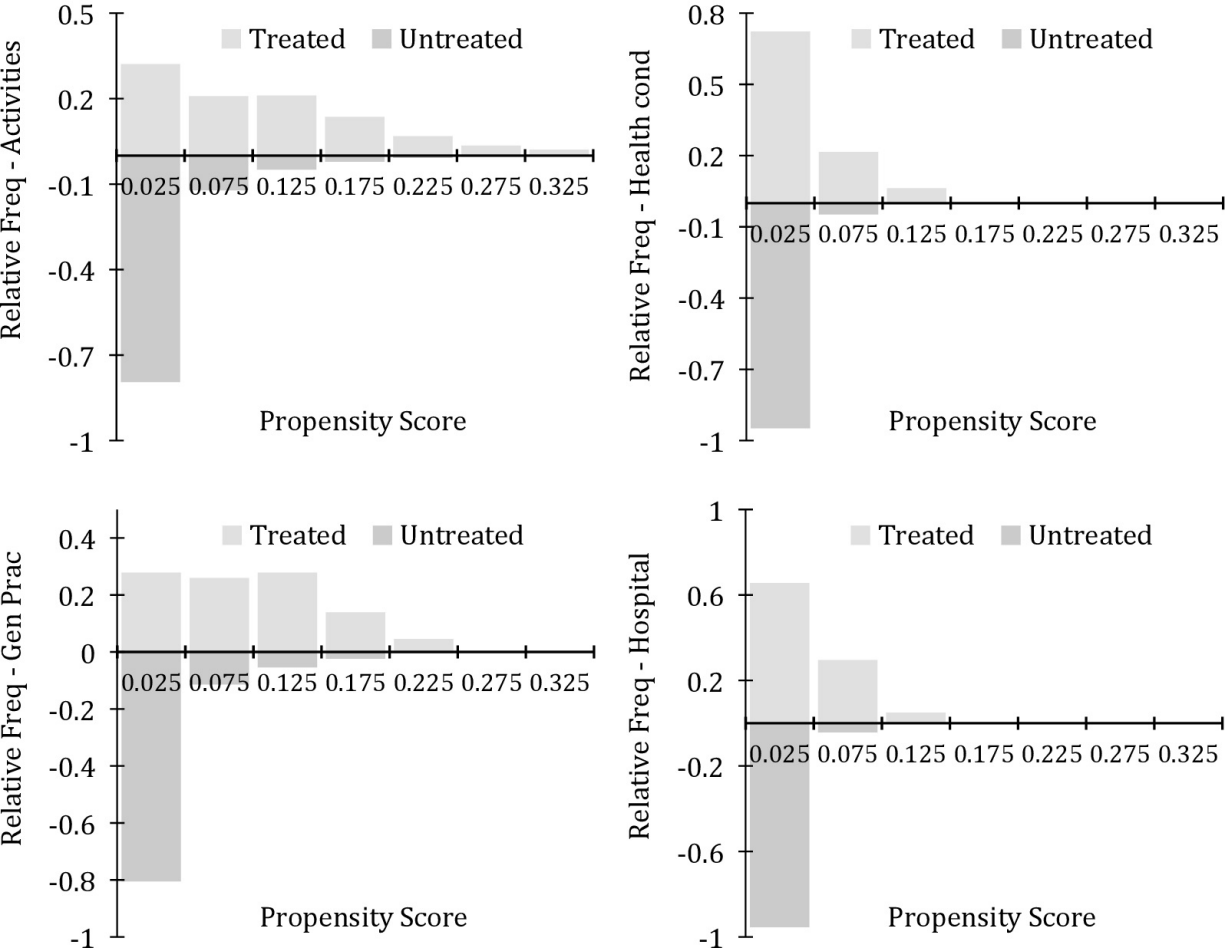
Common support histograms–healthcare shortfalls, mental condition ^a^. ^**a**^ The vertical axes give the relative frequencies for the propensity scores for each form of healthcare shortfall. The horizontal axes provide the numerical values of the scores. The top left panel represents help with home activities, the top right home healthcare, and the bottom panels give insufficient access to general practitioners and hospital services.

A standard tool for analysing the respective support structures of the propensity scores comes from non-parametric distributional plots. In Figs 1 and 2 we illustrate the distributions of the scores for each healthcare concept using histograms, where the distributions for treated individuals are depicted with positive bars in light grey, while untreated individuals are shown with negative bars in dark grey. Fig 1 shows that for physical health, over all four shortfall measures, the support of the treated individuals generally corresponds closely with the support of the untreated. This implies that for each individual in the treated group, there is a corresponding person in the untreated group such that suitable matches are available. Indeed the only instances where there are shortages of matches for treated individuals are for shortfalls in community care for health conditions for individuals with high propensity scores (over 0.5) and a lack of access to hospital services (propensities around 0.125). However it should be noted that our treated subsamples represent only from 1–10% of the full sample, and therefore there are many potential matches available for any treated observation. In all instances the full 100% of scores for treated observations had support on the distribution of values for the untreated.

Results for the mental health estimates are given in Fig 2. Again over the four forms of service shortfalls we see that the support range for treated individuals is similar to that for untreated and therefore appropriate matches can generally be made. For individuals with very high scores in the treatment groups there is in some cases a limited probability mass in the untreated group (e.g. for persons who require additional help with household activities and a lack of access to general practitioners). However as above, over all four models we find that the full sample of treated individuals share common support with the untreated. Thus while there tends to be fewer matching options when the propensity scores are high, individuals can still be appropriately linked across subgroups.

A second diagnostic tool comes from the covariate balance of the treated and untreated subgroups. If the PSM model is correctly specified then we do not expect to see significant differences in covariate distributions across individuals with the same propensity scores. Conversely if there are large differences in the covariates across the two groups the counterfactual created by the matching process may not be valid, which may in turn bias our results. For each estimation depicted in Table 4 we now consider the average percentage difference in means of the control variables between the matched subgroups. For comparability we also report the average percentage bias prior to matching. The results are given in Table 5.

**Table 5:** Mean percentage covariate bias–matched and unmatched samples ^a^.

	Help with Activates		Help with health condition		GP		Hospital	
	PH	MH	PH	MH	PH	MH	PH	MH
PSM NN *k* = 1	3.0	3.3	2.6	5.2	1.5	4.5	5.2	6.3
PSM NN *k* = 2	2.2	3.3	2.8	3.8	1.2	3.3	4.4	8.2
PSM NN *k* = 3	2.2	2.7	2.3	4.1	1.0	2.8	3.6	5.6
PSM *C = 0*.*005*	0.9	1.3	1.4	2.1	0.5	1.7	0.9	2.5
PSM *C = 0*.*01*	0.8	1.4	1.2	2.1	0.4	2.0	1.1	2.8
PSM Kernel	0.9	3.6	1.2	10.3	0.7	3.4	6.7	20.2
Prior	13.8	19.7	17.6	16.8	13.1	17.7	15.2	22.2

^**a**^ The columns provide the covariate bias estimates by inadequate service type by condition and the rows give the matching technique employed. Each estimate is given in terms of average percentage bias. The final row gives the bias in the treated and untreated groups prior to matching.

Results from Table 5 highlight the effectiveness of the matching algorithms in eliminating bias from the covariates over the matched samples. Prior to matching the average absolute bias across the treated subgroup relative to the untreated tended to lie between 13% and 22% (depending upon the form of shortfall) indicating that the reference and comparison groups differed substantially in all cases. After matching we see the degree of bias becoming greatly reduced, although the relative benefit varies by matching procedure. Looking at the first three rows we see that the NN methods tend to reduce the percentage bias to about 3–4% from the unmatched subsamples. While this represents a vast improvement the result is bettered by the caliper matches, which both reduced covariate bias to around 0–2.5% over the different shortfall service measures. The kernel estimates were in general also an improvement on the NN methods although the performance of this algorithm was extremely poor when dealing with cases when the treated group was very small relative to the untreated. For this reason we de-emphasize results based upon the kernel weights when looking at the effects of inadequate community care services and hospital services for persons with mental health problems.

## Discussion

This study indicated that ER presentation was associated with access to specific healthcare services and the type of disabling condition. These effects were notably large. Specifically, inadequate access to external medical services (GP and hospital services) increased ER rates for individuals with mental and physical conditions by about the same degree. In contrast, shortfalls in community care services in the form of organised services that assist with daily activities and health conditions significantly predicted ER visits for individuals with physical conditions, but not for persons with mental conditions. Taken at face value these results imply policy should focus more on improving the provision of external medical services in order to lower rates of ER presentation, and less community care, especially for persons with mental health conditions.

The links between ER use and inadequate access to external medical services were not surprising. Untreated physical conditions have the potential to rapidly deteriorate, and since physical conditions often accompany mental health conditions it is likely that comorbidity complications can lead to ER presentations. Also, mental health conditions are often associated with physical conditions related to neglect as dehydration, incorrect medication dosages or urinary tract infections which may result in a need for urgent medical attention.

The links between ER use and our community care variables are considerably less strong. Evidence shows that adequate and appropriate community care services by formal providers reduces institutionalisation rates among frail and disabled individuals [3, 28]. This supports our own findings which showed shortfalls in community care services significantly predicted ER visits for physical conditions, however the lack of a similar link for individuals with mental conditions was surprising. We offer several possible explanations for this finding.

Firstly, not all hospitals, including those in metropolitan areas, include a mental health unit. This means that people with a mental health condition are unlikely to visit the ER unless they possess a physical condition that needs urgent attention. Secondly, among individuals experiencing a mental health condition, there may be socio-demographic differences between those who receive medical services and those who receive community care services. Indeed, the vast majority of individuals receiving community care services consist of older cohorts, i.e. those aged 65 and over. There is a greater incidence of dementia or Alzheimer condition among older clients. Typically they require assistance with daily activities. In contrast, functioning individuals with non-age related mental health conditions such as schizophrenia, bipolar and depression are more likely to seek medical assistance from GPs or other health professionals to manage their condition. The sporadic episodes of these non-aged related mental health conditions can cause functioning adults to seek ER services.

Thirdly the decision to seek ER requires higher order thinking. The physical motivation to seek help may be lacking among those with a mental illness. For this reason, there is the possibility of people with a mental health condition not accessing the community services until they reach a crisis situation. For instance, when an individual is a danger to themselves or others, entry to ER will only occur via third party intervention. In contrast, those receiving assistance from GP or hospital services are likely to possess the cognitive ability to seek out a clinician for assistance (or have family support). Inadequately treated mental health conditions might be less ‘able’ to show up in ER, which could offset the increased risk of accident or emergency.

Patient behaviour is also an important consideration, especially if individuals experiencing a physical condition belong to the older age group. Many older people may prefer to visit the ER with the onset of an episode. People with severe mental illness may have ‘compliance issues’ around medication, which is where they may have exacerbations. Also, many in the community believe that their GP cannot perform certain procedures that are administered in an ER such as dealing with broken bones and stiches.

### Limitations

While in this paper we assume that unmet needs implies inadequate services, this may not always be the case. The inadequate access variable used in the analysis may also capture people’s preferences for ER services compared to other health services. For instance, public hospitals provide same day services on a greater range of diagnostic testing and health care services compared to the limited services provided by GPs.

The self-reported measure of unmet need for medical care may introduce bias. People requiring medical attention are more likely to have an actual experience of unmet need. In contrast, people with similar health issues who did not use services may have an unmet need which is not recognized. This can introduce reverse causation since people who use the ER are more likely to report a prior unmet need compared to their counterparts.

## Conclusion

The evidence we have regarding the shortfalls in medical and community care services and the type of condition that predict ER use shows that greater attention needs to be given to providing more flexible and appropriate access to health care services in the community setting. While it is well established that adequate medical care provided by GPs plays an important role in reducing ER visits, the link between ER visits and organised community care services that assist with daily activities and health conditions is poorly understood. This is surprising given the growing importance of community care as an integral part of the health sector of many western nations. Furthermore, the global trend towards deinstitutionalisation of mental health services warrants attention by researchers. Our study narrowed the knowledge gap in this area of research.

Among those experiencing a mental health condition, the high association of ER rates with inadequate medical services, combined with a lack of a similar association with inadequate community services is alarming. The explanation we find most plausible for this finding is that unless an individual is in physical rather than mental distress, crisis ER treatment is less often sought or unavailable, in which case more effort should be made to extend community care services to these individuals.

## References

[1] Lowthian J, Curtis A, Cameron P, Stoelwinder J, Cooke M, McNeil J. Systematic review of trends in emergency department attendances: an Australian perspective. Emergency Medicine Journal. 2011;28:373–7. 10.1136/emj.2010.099226 20961936

[2] Kelly A, Chirnside A, Curry C. An analysis of unscheduled return visits to an urban emergency department. New Zealand medical Journal. 1993;106:334–5. 8341474

[3] Bowles K, Naylor M, Foust J. Patient characteristics at hospital discharge and a comparision of home care referral decisions J Am Geriatric Society. 2002;50(2):336–42. 10.1046/j.1532-5415.2002.50067.x 12028217

[4] Australian Bureau of Statistics. 4839.0—Patient Experiences in Australia: Summary of Findings, 2013–14. Australian Bureau of Statistics,. 2014.

[5] Law C, Yip P. Acute care service utilization and the possible impacts of a user-free policy in Hong Kong. Hong Kong Medical Journal. 2002;8:348–53. 12376712

[6] Lee A, Lau F, Clarke H, Kam C, Wong P, Wong T, et al Factors associated with non-urgent utilization of Accident and Emergency services: a case-control study in Hong Kong. Social Science and Medicine. 2000;51:1075–85. 11005394 10.1016/s0277-9536(00)00039-3

[7] Taylor MJ, Horey D, Livingstone C, Swerissen H. Decline with a capital D: long-term changes in general practice consultation patterns across Australia. Medical Journal of Australia. 2010;193 (2):80–3. 20642408 10.5694/j.1326-5377.2010.tb03804.x

[8] Callen JL, Blundell L, Prgomet M. Emergency department use in a rural Australian setting: are the factors prompting attendance appropriate?. Aust Health Rev. 2008;32(4):710–9. 18980567 10.1071/ah080710

[9] Kravet SJ, Shore AD, Miller R, Green GB, Kolodner K, Wright SM. Health care utilization and the proportion of primary care physician. The American Journal of Medicine. 2008;121(2):142–8. 10.1016/j.amjmed.2007.10.021 18261503

[10] Padgett D, Brodsky B. Psychosocial factors influencing non-urgent use of the emergency room: A review of the literature and recommendations for research and improved service delivery Social Science and Medicine. 1992;35(9):1189–97. 1439937 10.1016/0277-9536(92)90231-e

[11] Hadley TR, McGurrin MC, Pulice RT, Holohean EJ. Using fiscal data to identify heavy service users. Psychiatr 1990;Q: 61:41–8. 10.1007/BF01065163 2201053

[12] Haywood TW, Kravitz HM, Grossman LS, Cavanaugh JL, Davis JM, Lewis DA. Predicting the “revolving door” phenomenon among patients with schizophrenic, schizoaffective, and affective disorders. Am J Psychiatry. 1995;152:856–61. 10.1176/ajp.152.6.856 7755114

[13] Klijakovic M, Allan BC, Reinker J. Why skip the General Practice and go to the Accident and Emergency Department?. NZ Medical Journal. 1981;93:49–52. 6944635

[14] Mechanic D. Correlates of physician utilization J Hlth Soc Behav 1979;20:387. 317291

[15] Reder S, Hedrick S, Guihan M, Miller S. Barriers to home and community-based service referrals: The physician's role Gerontology and Geriatrics Education. 2009;30:21–33. 10.1080/02701960802690241 19214844

[16] Wright D, Ricketts T. The road to efficiency? Re-examining the impact of the primary care physician workforce on health care utilization rates. Social Science and Medicine. 2010;70. 10.1016/j.socscimed.2010.02.043 20385438

[17] Liaw S, Hill T, BRyce H, Adams G. Emergency and primary care at a Melbourne hospital: reasons for attendance and satisfaction. Australian Health Review. 2001;24(2):120–34. 11496454 10.1071/ah010120a

[18] Ingram D, Clarke D, Murdie R. Distance and the decision to visit an emergency department. Social Science and Medicine. 1978;12:55–62. 644349

[19] Holmes D. New initiative aims to tackle shortfalls in mental health crisis care. The Lancet Psychiatry. 2014;1(1):15–6. 10.1016/S2215-0366(14)70267-6 26360393

[20] Survey Finds Shortfalls in the Quality of Mental Health Care for Older Americans. Psychiatric Services. Psychiatric Services. 2013;64(2). 10.1176/appi.ps.642news1 23370630

[21] Vecchio N, Stevens S, Cybinski P. Caring for People with a Mental Disability at Home. Carers’ Perceptions of Service Provision Community Mental Health. 2008;44(2):125–34. 10.1007/s10597-007-9111-x 18165898

[22] Tang D, Gilligan A, Romero K. Economic burden and disparities in health care resource use among adult patients with cardiac arrhythmia Applied Health Economics and Health Policy. 2014;12:59–71. 10.1007/s40258-013-0070-9 24311201

[23] Australian Bureau of Statistics. Disability Ageing and Carers 2012 Basic. In: Australian Bureau of Statistics, editor. Canberra 2012.

[24] Andersen R, Newman J. Societal and individual determinants of medical care in the United States. Milbank Q 1973;51(95). 4198894

[25] White H. A Heteroskedasticity-Consistent Covariance Matrix Estimator and a Direct Test for Heteroskedasticity. Econometrica. 1980;48(4):817–38.

[26] Austin PC. An Introduction to Propensity Score Methods for Reducing the Effects of Confounding in Observational Studies Multivariate Behavioral Research. 2011;46(3):399–424. 10.1080/00273171.2011.568786 21818162 PMC3144483

[27] Leuven E, Sianesi B. PSMATCH2: Stata module to perform full Mahalanobis and propensity score matching, common support graphing, and covariate imbalance testing 2003.

[28] Productivity Commission. Trends in Aged Care Services: some implications Commission Research Paper In: Productivity Commission, editor. Canberra 2008.

